# From safer alternative to public health crisis: the unintended consequences of e-cigarettes

**DOI:** 10.1097/MS9.0000000000005026

**Published:** 2026-04-22

**Authors:** Syed Shahmeer Ahmed, Farhan Ahmed, Abdul Ghafoor Qureshi, Ahmed Asad Raza, Abedin Samadi

**Affiliations:** a Department of Medicine, Karachi Medical and Dental College, Karachi, Pakistan; b Department of Medicine, Jinnah Sindh Medical University, Karachi, Pakistan; c Department of Medicine, Kabul University of Medical Sciences Abu Ali Sina, Kabul, Afghanistan

**Keywords:** e-cigarettes, nicotine dependence, public health regulation, respiratory health, vaping

## Abstract

Electronic cigarettes (e-cigarettes) were introduced as a harm-reduction alternative to combustible tobacco, offering nicotine delivery without combustion-related toxicants. However, their rapid global adoption has produced complex and unintended public health consequences. This editorial examines the evolving evidence on the safety, respiratory effects, and population-level impact of e-cigarettes. Early safety assumptions were largely based on ingestion toxicology rather than inhalation data, and many products entered the market without comprehensive preclinical or long-term human safety evaluation. Emerging epidemiological studies associate e-cigarette use with increased respiratory symptoms, while mechanistic research demonstrates airway inflammation, oxidative stress, and exposure to ultrafine particles, aldehydes, and metal contaminants. The 2019 outbreak of e-cigarette or vaping-associated lung injury lighted systemic regulatory vulnerabilities in the oversight of inhaled consumer products. In parallel, highly efficient nicotine delivery systems and youth-targeted product features have contributed to rising nicotine dependence among adolescents, shifting the public health burden. While some adult smokers may benefit from complete substitution, the net population effect remains uncertain when balanced against youth initiation and dual use. We argue that harm-reduction technologies require stronger regulatory frameworks, transparent risk communication, rigorous pre- and post-market evaluation, and youth-protective policies. A recalibrated, evidence-driven approach is essential to ensure that harm reduction reduces – rather than redistributes – health risk.

## Introduction

Electronic cigarettes (e-cigarettes) were introduced in the early 2000s as a harm-reduction innovation aimed at one of public health’s most persistent challenges: combustible tobacco use. By delivering nicotine without combustion, these devices were promoted as a potentially safer alternative for adult smokers. Two decades later, however, e-cigarettes represent a far more complex public health challenge than initially anticipated. The 2019 outbreak of e-cigarette or vaping-associated lung injury (EVALI), which resulted in thousands of hospitalizations and 68 confirmed deaths in the United States, marked a turning point in public awareness and regulatory scrutiny[1]. Although EVALI was primarily linked to vitamin E acetate in illicit tetrahydrocannabinol products, the outbreak exposed a broader systemic failure: the rapid diffusion of inhaled nicotine technologies in the absence of robust pre-market safety evaluation. Critically, comprehensive preclinical inhalation toxicology studies and long-term human safety trials were largely absent or limited prior to the widespread commercial adoption of these products, reflecting gaps in regulatory oversight at the time of market entry[2]. What began as a smoking cessation concept has evolved into a large and rapidly expanding global commercial market[2]. While some adult smokers may reduce exposure to combustion-related toxicants by switching to e-cigarettes, evidence increasingly indicates that this transition has been accompanied by new patterns of nicotine dependence and emerging concerns about respiratory health. This editorial argues that these outcomes reflect structural weaknesses in how harm-reduction innovations are regulated and communicated, highlighting the need for a recalibration of public health policy and perception.

## The illusion of safety: when assumption replaced evidence

The rapid rise of e-cigarettes was driven by a compelling narrative of relative safety. Sleek device design, appealing flavors, and aggressive marketing reframed vaping as a clean, modern lifestyle choice distinct from smoking[3]. This transformation occurred during a period of limited regulatory oversight, allowing products to reach consumers without toxicological scrutiny comparable to that required for pharmaceutical nicotine replacement therapies^[^2,3^]^.

Critically, safety was inferred rather than demonstrated. Core e-liquid components – propylene glycol, vegetable glycerin, nicotine, and thousands of flavoring agents – were considered safe for ingestion, yet their chronic inhalation was largely unstudied[4]. Meanwhile, product evolution accelerated. Nicotine salt formulations enabled rapid systemic nicotine delivery with reduced throat irritation, increasing addiction potential while lowering barriers to initiation among inexperienced users^[^3,4^]^. This convergence of technological innovation and regulatory lag created an environment in which commercial scalability preceded scientific certainty.

## Respiratory effects: emerging signals and persistent uncertainty

A growing body of epidemiological evidence associates e-cigarette use with increased prevalence of respiratory symptoms, including cough, wheeze, chest tightness, and shortness of breath, particularly among frequent users[5]. Most epidemiological studies examining respiratory outcomes are cross-sectional and therefore cannot establish causal relationships. These analyses are also vulnerable to confounding from prior cigarette smoking, cannabis use, or environmental exposures. Nevertheless, the consistency of associations across diverse populations suggests that the potential respiratory effects of e-cigarette aerosol warrant continued investigation. Evidence regarding asthma outcomes remains mixed; some studies report no significant association with asthma exacerbations, whereas others, primarily based on survey data, suggest an increased symptom burden, particularly among adolescents^[^5,6^]^.

Mechanistic data provide biological plausibility. E-cigarette aerosols contain ultrafine particles, reactive aldehydes such as formaldehyde, and metal nanoparticles from heating elements, all of which can deposit deep within the alveoli and induce oxidative stress and inflammation^[^4,7^]^. Although long-term data on chronic lung disease are limited, early findings raise concern that repeated exposure may alter airway physiology.

The EVALI outbreak, though not representative of standard nicotine e-cigarette use, remains instructive. It demonstrated how an unregulated inhalation product ecosystem can rapidly produce severe pulmonary toxicity when safety controls fail^[^1,8^]^. EVALI should be understood not as evidence that all vaping causes acute lung injury, but as a sentinel event underscoring the vulnerability of the respiratory system to novel inhaled chemicals introduced without adequate oversight.

## Youth vaping and nicotine dependence

Parallel to respiratory concerns is the rise of e-cigarette use among adolescents. In the United States, national surveillance data indicate that past-month e-cigarette use among high school students increased from approximately 1.5% in 2011 to over 27% by 2019, before declining modestly following regulatory action[9]. Despite this decline, prevalence remains substantially higher than that of combustible cigarette use, which continues at historic lows[10].

This pattern reflects exposure to highly efficient nicotine delivery systems rather than benign experimentation. Modern e-cigarettes can deliver nicotine concentrations comparable to cigarettes, facilitating rapid dependence[9]. Nicotine exposure during adolescence has been associated with alterations in cognitive development, reward processing, and impulse control, supported by neurobiological studies demonstrating nicotine-induced changes in synaptic plasticity and neural circuitry during brain maturation^[^9–12^]^. Longitudinal studies demonstrate increasing frequency of use and dependence among adolescent vapers, with a subset progressing to daily consumption[10].

Debate persists regarding whether e-cigarettes serve as a gateway to combustible smoking. While youth combustible cigarette smoking rates have not rebounded, the absence of increased smoking does not diminish the public health significance of widespread nicotine dependence among adolescents who might otherwise have remained nicotine-free^[^13,14^]^. For youth, the appropriate comparator is not combustible tobacco but no nicotine exposure at all.

Although much of the surveillance evidence comes from the United States, rising adolescent e-cigarette use has also been reported internationally. Reports from the World Health Organization and national surveillance programs in Europe and Australia indicate increasing youth experimentation with vaping products, highlighting that this is a global public health concern rather than a U.S.-specific phenomenon[10].

## Rethinking harm reduction at the population level

Evidence suggests that adult smokers who completely switch from cigarettes to e-cigarettes may reduce exposure to certain toxicants. However, public health policy must be guided by population-level effects rather than individual benefit alone. When a product simultaneously offers potential benefit to some smokers, while fostering nicotine dependence among youth and non-smokers, its net public health impact becomes uncertain. The common framing of e-cigarettes as “less harmful than cigarettes” is context-dependent and misleading when applied to populations for whom smoking would not otherwise have occurred.

## A path forward

A responsible approach to e-cigarettes should rest on five principles: (1) transparent risk communication distinguishing adult smoking substitution from youth initiation risk; (2) prioritization of youth protection through restrictions on flavors, marketing, and access; (3) rigorous pre-market and post-market evaluation of inhaled products; (4) independent, long-term research on chronic respiratory, cardiovascular, and neurodevelopmental outcomes; and (5) coordinated global regulation to prevent harm displacement to low- and middle-income countries.

## Conclusion

The trajectory of e-cigarettes illustrates the risks of deploying harm-reduction technologies without sufficient evidence or safeguards. While offering potential benefits for some adult smokers, e-cigarettes have generated new and unevenly distributed harms. Innovation without adequate evidence risks becoming experimentation on a population scale. Given the transnational nature of the vaping industry and digital marketing ecosystems, regulatory strategies must also operate at an international level to prevent harm displacement across borders. Future public health strategies must ensure that harm reduction reduces harm for all not by shifting it to those least able to bear the cost.

To ensure methodological transparency and ethical compliance in manuscript preparation, we adhered to the recently developed TITAN (Transparency in the Reporting of Artificial Intelligence) 2025 guidelines, which outline standards for responsible AI use in biomedical writing and publishing[15].

## Data Availability

No datasets were generated or analyzed during the current study.
